# Randomized controlled trial to evaluate the effects of ethyl-2-cyanoacrylate on pain intensity and quality of life in head and neck cancer patients suffering from cetuximab-induced rhagades during radioimmunotherapy: the support trial

**DOI:** 10.1186/1471-2407-14-270

**Published:** 2014-04-17

**Authors:** Karin Potthoff, Gregor Habl, Thomas Bruckner, Christian Suppan, Jessica Hassel, Dirk Jäger, Martin Indorf, Juergen Debus

**Affiliations:** 1 Department of Radiation Oncology, National Center for Tumor Diseases, University of Heidelberg, Heidelberg, Germany; 2 Institute for Medical Biometry and Informatics, University of Heidelberg, Heidelberg, Germany; 3 Department of Medical Oncology, National Center for Tumor Diseases, University of Heidelberg, Heidelberg, Germany; 4 Department of Dermatology, National Center for Tumor Diseases, University of Heidelberg, Heidelberg, Germany; 5 iOMEDICO AG, Freiburg, Germany

## Abstract

**Background:**

Cetuximab is a chimeric monoclonal antibody against the epidermal growth factor receptor (EGFR). Skin reactions are the most common side effects of cetuximab. Rhagades of the tips of the fingers and toes, the heels and especially the interphalangeal joints are one of the most frightening and painful dermatological side effects that may develop from EGFR-inhibitor therapy. Rhagades are characterized by pain, severe tenderness and poor healing response. They are challenging to treat. Thus, rhagades often poses the most significant threat to the quality of life (QoL) for these patients. Ethyl-2-cyanoacrylate (ECA), an ethyl ester of the 2-cyano-2-propenoic acid, is often used as adhesive in a variety of different work settings in industry, i.e. as a component in nail-care products such as nail glue. In addition, ECA is used for various medical indications, such as for liquid bandages and for suture-less surgery. Wound healing can be accelerated with ECA. The purpose of the SUPPORT trial is to investigate the efficacy of ECA for the treatment of cetuximab-induced rhagades and to assess the clinical usefulness of the SUPO score, a new classification system for rhagades induced by EGFR-inhibitor therapy.

**Methods/Design:**

The SUPPORT trial is an open-label, prospective, randomized, national multicenter intervention study to evaluate the effectiveness of ECA versus the standard treatment of each institution on the pain intensity and QoL in patients with locally advanced head and neck cancer suffering from painful cetuximab-induced rhagades during radioimmunotherapy. Primary endpoint is the assessment of the pain intensity 24 hours after application of ECA or the standard treatment quantified by the visual analogue scale (VAS). Secondary endpoints are the evaluation of QoL assessed by the EORTC-QoL-C30 questionnaire and the Dermatological Life Quality Index (DLQI).

**Discussion:**

During treatment with EGFR inhibitors it is necessary to recognize and manage side effects promptly to assure better patient QoL. The SUPPORT trial is the first randomized clinical trial evaluating a new treatment option for painful cetuximab-induced rhagades. Furthermore, the new SUPO score will be prospectively assessed in terms of clinical usefulness for classification of EGFR inhibitor-induced rhagades.

**Trial registration:**

Current Controlled Trials NCT01693159.

## Background

Cetuximab is a chimeric monoclonal antibody against the epidermal growth factor receptor (EGFR). It has shown clinical activity against a variety of malignancies [1-5]. In head and neck cancer cetuximab is approved in combination with radiotherapy as a curative treatment option for patients with locally advanced squamous cell carcinoma of the head and neck (LASCCHN). In the pivotal phase III trial published by Bonner et al., a radioimmunotherapy with cetuximab resulted in a higher response rate, an improvement of the duration of locoregional control and an increased rate of 5-year overall survival [6-9]. Cetuximab, as with the entire class of anti-EGFR inhibitors is associated with a high prevalence of dermatological side effects [10-15]. Commonly experienced dermatological side effects include acneiform rash, hair changes, enhancement of radiation-induced dermatitis, pruritus, mucositis, xerosis cutis, rhagades and paronychia. While acneiform rash is the most common side effect during the first weeks of application of the monoclonal antibody cetuximab, xerosis of the skin and xerosis-associated rhagades usually develop after at least 5 to 6 weeks of treatment with an anti-EGFR inhibitor such as cetuximab [15-17]. In the European literature fissures of the skin and skin cracking are termed rhagades [18]. Rhagades of the fingertips and toes, of the palms or knuckles, the heels, the soles and especially of the interphalangeal joints are one of the most frightening and painful dermatological side effects that may develop as late phase skin reactions from EGFR-inhibitor therapy [19]. Rhagades occur in about 15% to 25% of all patients treated with an EGFR-inhibitor and are characterized by pain, severe tenderness and poor healing tendency. They can be very painful and, furthermore, may create a risk for local or systemic infection [16,17]. All of those dermatological toxicities including cetuximab-induced rhagades have often led to reduction or even cessation of an effective anticancer therapy and they have been shown to decrease patients' quality of life (QoL) significantly. Activities of daily living (ADL) may be impaired due to skin reactions, especially due to anti-EGFR induced painful rhagades. Whereas prevention and treatment recommendations for cetuximab-induced acneiform rash are well established today and have been published from several research groups recently [13-24], treatment recommendations for the treatment of cetuximab-induced painful rhagades are only reported anecdotally [15-18]. Lacouture et al. published general recommendations for the prevention and treatment of rhagades recently based on their own expert opinion. The individual recommendations for prevention of rhagades include the wearing of protective footwear or covering the fingertips to avoid friction; for treatment the authors recommended the topical application of thick moisturizer, zinc oxide creams, propylene glycol 50% solution, salicylic acid 10% ointment, steroid tapes and hydrocolloid dressings or liquid glues like cyanoacrylate preparations to keep the rhagades from worsening [17]. Limited evidence also supports the use of silver nitrate or potassium permanganate foams and topical antibiotics [17]. Oral antibiotics, however, may be necessary if infection of the rhagades occurs and worsens despite topical treatment. Randomized clinical trials assessing the prevention or treatment of EGFR-induced rhagades, however, have not been performed so far. No published data are available supporting prevention or treatment recommendations for those rhagades. Thus, evidence-based treatment recommendations for anti-EGFR induced painful rhagades do not exist. All recommendations are based on individual observation, case studies and expert opinion. Another problem is that for those rhagades typically seen during and after anti-EGFR treatment no suitable classification system or scoring system is available. The NCI CTCAE criteria do not comprise a useful scoring system for this type of side effects [25]. Thus, an appropriate scoring system is warranted to classify the EGFR inhibitor induced rhagades and to allow rational treatment decisions based on a standardized clinical scoring system.

Liquid glues such as ethyl-2-cyanoacrylate (ECA), an ethyl ester of the 2-cyano-2-propenoic acid, are often used as adhesive in a variety of different work settings in industry, e.g. as a component in nail-care products such as nail glue. Besides, ECA is commonly used for various medical indications, especially for the treatment of wounds, e.g. for liquid bandages in children and for suture-less surgery. It is a colorless liquid with low viscosity at normal room temperature and it polymerizes rapidly in the presence of moisture. Wound healing can be accelerated with ECA [12,17,26-28]. ECA is suitable for the treatment of rhagades due to the direct proportional influence of the OH-group concentration of ECA on the age hardening velocity which occurs within a spit second [17]. Sealing the cracks with ECA may also help to relieve pain [17,26-28]. Furthermore, applying ECA leads to wound closure and to an effective germ barrier which may lower the rate of secondary wound infections [29]. Dermatological toxicities, especially rhagades induced by EGFR inhibitors, critically affect patients’ health-related quality of life and, as a consequence, the dose intensity of EGFR inhibitors and, thus, the effectiveness of antineoplastic regimens. There are several patients known from observation who did not receive their planned course of anticancer treatment due to severe cutaneous side effects and who, therefore, had a poorer outcome of their disease. Due to the missing data from clinical trials on the prevention and the management of EGFR-inhibitor induced rhagades, the crucial impact of rhagades on the patient’s wellbeing and QoL and the adherence to anticancer treatment, prospective, randomized, controlled clinical trials are warranted to evaluate treatment options for EGFR-inhibitor induced rhagades, which hopefully may allow the establishment of evidence-based prevention and treatment guidelines.

The SUPPORT trial is a prospective, open-label, randomized, controlled intervention trial exploring the efficacy of ethyl-2-cyanoacrylate for the treatment of painful cetuximab-induced rhagades compared to the standard treatment of each institution. In addition, the clinical usefulness of the SUPO score, a new classification system for rhagades induced by EGFR-inhibitor therapy, will be assessed and validated in a clearly defined patient cohort.

## Methods and design

### Study objectives

The purpose of the SUPPORT trial is to evaluate the effect of topical applied ECA compared to standard treatment on the pain intensity and quality of life in patients suffering from painful cetuximab-induced rhagades during cetuximab-based radioimmunotherapy for LASCCHN. Focus of the analysis is to evaluate a superiority of ECA compared to any standard treatment used in the participating institutions concerning pain relieve and improvement of QoL.

### Primary objective

Primary endpoint is the assessment of the pain intensity 24 hours after application of ECA or the standard treatment of each institution quantified by the visual analogue scale (VAS).

### Secondary objectives

Secondary endpoints are the pain intensity assessed 5 to 7 days after application of ECA or the standard treatment of each institution quantified by the VAS and the evaluation of QoL assessed by the EORTC-QoL-C30 questionnaire and the Dermatological Life Quality Index (DLQI) 5 to 7 days after application of ECA or the standard treatment of each institution. Furthermore, a photo documentation of the rhagades will be performed on baseline, 24 hours after first application of ECA or standard treatment and 5 to 7 days later. Furthermore, the SUPO score will be used at screening, baseline, 24 hours and 5 to 7 days after application of the specific treatment for diagnostics as well as for the assessment of clinical response. The SUPO score is shown in Table 1.

**Table 1:** SUPO Score for the classification of rhagades

Grade 1:	Rhagades without clinical symptoms
Grade 2:	Painful rhagades
	Grade 2a: Moderate pain, no impairment of activity in the daily routine (ADL)
	Grade 2b: Severe pain and impairment of the activities of daily living (ADL)
Grade 3:	Painful, deep and spontaneously bleeding rhagades
Grade 4:	Superinfection of the rhagades (detection of bacterial growth)
	Grade 4a: Local infection
	Grade 4b: Systemic infection
Grade 5:	Death due to complications of the rhagades

### Design/Randomization

**Figure 1:**
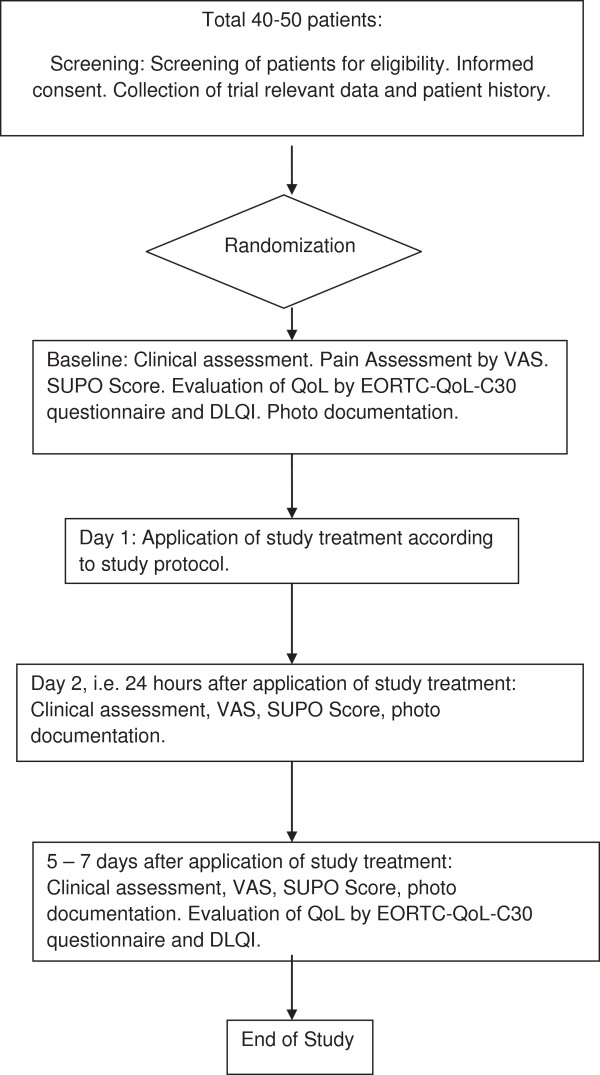
Flow chart of the SUPPORT trial.

SUPPORT is a randomized controlled intervention study, in which approximately 40-50 patients will be randomized 1:1 to receive ECA or the standard treatment of the institution (Figure 1). Allocation of patients to either treatment group is concealed by using a centralised randomisation procedure with a computer generated list produced by an independent research organisation, i.e. iOMEDICO AG, Freiburg, Germany. The participating study center will complete the randomization form and fax the page to iOMEDICO AG. An iOMEDICO-employee not involved in the project management, monitoring or data management of the study will assign the treatment arm according to the randomization list and will forward this information to the study center. The randomization list will be kept in safe and confidential custody at iOMEDICO AG.

#### Setting

The SUPPORT trial will be performed as a multicenter study according to the § 23b of the German Medicinal Devices Act (MPG). The study setting is national, with approximately 40 sites in Germany. The SUPPORT trial is designed by the study initiators of the Department of Radiation Oncology and the Department of Medical Oncology at the National Center for Tumor Diseases of the University of Heidelberg Medical Center. In view of the multimodal nature of the trial, all investigators are experienced oncologists in the fields of radiation oncology and medical oncology.

## Patient selection: inclusion and exclusion criteria

Patients with the diagnosis of LASCCHN and treated with primary definitive cetuximab-based radioimmunotherapy within the HICARE clinical trial protocol [30] will be evaluated and screened for the participation in the SUPPORT trial at the time when they develop cetuximab-induced rhagades. All patients fulfilling the inclusion and exclusion criteria will be informed about the study.

### Inclusion criteria

Patients meeting all of the following criteria will be considered for admission to the trial:

– LASCCHN and participation in the HICARE-phase-IV-trialCetuximab-induced painful rhagades, i.e. SUPO Score 2-3 (see Figure 1)

– Compliance to the photo documentation

– Ability of subject to understand character and individual consequences of the clinical trial

– Written informed consent

### Exclusion criteria

Patients presenting with any of the following criteria will not be included in the trial:

– Cetuximab-induced rhagades without any pain, i.e. SUPO Score 1 (see Figure 1)

– Cetuximab-induced rhagades, SUPO Score 4, i.e. superinfection of the rhagades (see Figure 1)

– Patients not being enrolled in the HICARE trial

– Substance misuse, psychoactive substance abuse or psychological/social conditions leading to a decreased patients’ compliance with possible bad influence to the results of the study

– Known allergic reaction to ethyl-2-cyanoacrylate (ECA)

## Treatment schedule

After achievement of the written informed consent patients will be 1:1 randomized in the experimental arm or the control arm. All patients will receive a specific treatment for their painful rhagades. In the experimental arm patients will be topically treated with the liquid glue ethyl-2-cyanoacrylate (ECA) whereas in the control arm patients will obtain the standard therapy of the institution. All patients will be assessed for pain, SUPO Score and QoL after 5 to 7 days, respectively.In case of insufficient response and absent clinical benefit of standard treatment of the institution in the control arm 5 to 7 days after begin of treatment a cross over to the experimental group can be performed due to ethical reasons. No statistical influence on any endpoint will be expected due to the cross over design. End of study is 5 to 7 days after first application of ECA or the standard treatment of the institution. No further follow-up visits are scheduled within the trial. The last patient included into the study will be followed for exactly 5 to 7 days after start of treatment. This is considered the final study visit. The overall duration of the trial is expected to be approximately 24 months. All participants have the right to drop out the trial at any time.

## Assessment of safety parameters

Safety and toxicity of the study treatment will be evaluated by clinical examination. The International Common Terminology Criteria for Adverse Events (CTCAE) version 4.02 will be used for toxicity and adverse event reporting. A copy or the CTCAE can be accessed from the CTEP home page: http://ctep.cancer.gov/protocolDevelopment/electronic_applications/ctc.htm.

## Statistical methods

### Study hypothesis

The study is designed to demonstrate a superiority of ECA compared to standard treatment of each institution for treatment of cetuximab-induced painful rhagades.

### Statistical calculations for trial sample size

The primary endpoint of this trial is the change of pain 24 hours after therapy compared to pain before therapy, and pain is measured using a visual analog scale (VAS). Assuming a mean difference in pain reduction of 20% and a standard deviation of 20%, 17 evaluable patients per group are needed to detect this difference with a power of 1-β = 80% and a level of significance α = 5% when applying a t-test. It can be expected that the actual power of the test is higher when applying an analysis of covariance with pain (VAS) before therapy as a continuous covariate. Assuming a drop-out rate of 15%, another 3 patients have to be randomized in each of the two treatment groups to get a total of n = 20 patients per group.

### Statistical methods

The confirmatory analysis is performed on the basis of an intention-to-treat (ITT) population and with respect to ITT principles. Additional analysis will be conducted on the per-protocol population.

Descriptive statistics for continuous parameters and scores include the number of non-missing observations, mean, standard deviation, median, minimum and maximum, performed for the treatment groups and overall. The description of categorical variables (ordinal or nominal) includes the number and percentage of patients belonging to the relevant categories in the trial population as well as to each treatment group.

The primary efficacy endpoint is the difference of the pain score assessed by VAS 24 h after begin of treatment. The underlying two sided null-hypothesis is that both interventions lead to similar means of the VAS pain in both intervention groups 24 hours after therapy.

$$H_{0}:µ1–µ2=0$$

The alternative hypothesis is that any intervention performs better than the other:

$$H_{A}:µ1–µ2\neq 0$$

A confirmatory intention to treat analysis (2-sided test), including all patients as randomized, will be performed on the mean differences in the VAS pain values between the two treatment groups. Analysis of covariance (ANCOVA) techniques will be used to detect possible treatment effects, with VAS pain score before therapy as a continuous covariate.

All patients will be included for analysis for secondary endpoints treated at least once with the study treatment. Secondary endpoints will be analyzed in an exploratory fashion, using appropriate statistical methods based on the underlying distribution of the data.

Graphical methods including scatter plots and box-plots will be used to visualize possible correlations between continuous parameters and differences between intervention groups.

All analyses will employ SAS Version 9.1.

### Interim analyses and stopping rules

No formal interim analysis is planned. Patients whose study therapy will be stopped due to toxicity will be considered treatment failures. In case of safety concerns, e.g. toxic events CTCAE grade 3 or more in more than 5% of patients, the principal investigator has to decide on early study termination.

## Data handling, storage and archiving of data

According to the §13 of the German GCP-Regulation all important trial documents will be achieved for at least 10 years after the trial termination.

According to the §28c of the German X-ray Regulation (RöV) and the §87 of the German Radiation Protection Regulation (StrlSchV) the informed consent forms including patients consent for trial participation, application of irradiation and data transmission to the competent authority will be achieved for at least 30 years after the trial termination.

The Clinical Trials Center of the Department of Radiation Oncology will be responsible for archiving all relevant data.

## Good clinical practice (GCP)

The procedures set out in this trial protocol, pertaining to the conduct, evaluation, and documentation of this trial, are designed to ensure that all persons involved in the trial follow the guidelines of Good Clinical Practice (GCP) and the ethical principles described in the applicable version of the Declaration of Helsinki (2008 Version of the Declaration of Helsinki, adopted at the 59th WMA General Assembly, Seoul, October 2008), as well as in accordance with the “Berufsordnung für Ärztinnen und Ärzte” in the most recent version. The trial will be carried out in adherence to local legal and regulatory requirements.

## Ethics, informed consent and legal aspects

A positive Ethics Vote was obtained from the independent Ethics Committee of the Medical Faculty of the University of Heidelberg, Germany (S-542/2010) and the local ethics committee of every participating site. The SUPPORT trial is registered at http://www.clinicaltrials.gov website, number NCT01693159.

Participation of a patient in this study is voluntary. Before being admitted to the clinical trial, the subject must consent to participate after the nature, scope, and possible consequences of the clinical trial have been explained in a form understandable to him or her. The subject must give written informed consent for study participation. A subject may voluntarily discontinue participation in this study at any time at their own request. Before study entry, patients will be informed by the written information brochure as well as orally about the planned procedures within this study, especially about potential benefits or potential risks. Informed consent will be documented by the patient's signature on the informed consent form.

In case of withdrawal of a subject at his/her own request, the reason should be asked for as extensively as possible and should be documented. All data acquired within this study will be allowed for further evaluation and inclusion into the final analysis due to written informed consent and data privacy statement.

The data obtained in the course of the trial will be treated pursuant to the Federal Data Protection Law (Bundesdatenschutz- bzw. Landesdatenschutzgesetz, BDSG, LDSG).

## Discussion

EGFR inhibitors such as cetuximab are associated with a unique group of class-specific cutaneous toxicities. Rhagades are one of the most painful and most dreaded complications from EGFR inhibitor therapy Currently, neither a scoring system for cetuximab-induced rhagades for diagnostics nor a standard of care for the treatment of the rhagades is established. Prevention and management of EGFR-inhibitor-related rhagades, however, is critical to maintain patients’ health-related quality of life and dose intensity of antineoplastic regimens as the pain, the discomfort and the reduced QoL caused by the rhagades can reduce compliance with anti-EGFR therapy. For this reason, exact diagnosis and appropriate treatment are very important. The SUPPORT trial is the first randomized, controlled clinical trial evaluating a new treatment option for painful cetuximab-induced rhagades. The main goal of this study is to show a superiority of ECA compared to common standard treatments for patients with LASCCHN developing painful rhagades during combined radioimmunotherapy with the monoclonal EGFR-antibody cetuximab. Since there is no scoring system for rhagades described elsewhere, the new SUPO score will be prospectively assessed in terms of clinical usefulness for the classification of EGFR inhibitor induced rhagades. An effective management of the painful rhagades is essential to assure a better quality of life for the patients, to allow a better adherence to cancer therapy and to avoid interruptions or even discontinuation of antineoplastic treatment associated with poor outcome. The results of the SUPPORT trial may help to define an evidence-based treatment approach for EGFR-inhibitor induced rhagades in the near future.

## Abbreviations

ADL: Activities of daily living; DLQI: Dermatological life quality index; ECA: Ethyl-2-cyanoacrylate; EGFR: Epidermal growth factor receptor; GCP: Good clinical practice; LASCCHN: Locally advanced sqamous cell carcinoma of the head and neck; VAS: Visual analogue scale; QoL: Quality of life.

## Competing interests

The authors declare that they have no financial or non-financial competing interests.

## Authors’ contributions

KP, GH, CS, DJ and JD have developed the study concept. KP and CS wrote the study protocol and obtained ethics approval. KP, GH, JH and JD provide patient care. TB performed the statistical calculations and will be responsible for the final statistical analysis. MI represents iOMEDICO AG, the CRO responsible for randomization, eCRF programming and logistics concerning QoL questionnaire. All authors read and approved the final manuscript.

## Pre-publication history

The pre-publication history for this paper can be accessed here:

http://www.biomedcentral.com/1471-2407/14/270/prepub
